# Association of incident dialysis modality with mortality: a protocol for systematic review and meta-analysis of randomized controlled trials and cohort studies

**DOI:** 10.1186/s13643-019-0972-1

**Published:** 2019-02-19

**Authors:** Mark R. Marshall, Chun-Yuan Hsiao, Philip K. Li, Masaaki Nakayama, S. Rabindranath, Rachael C. Walker, Xueqing Yu, Suetonia C. Palmer

**Affiliations:** 1 School of Medicine, Faculty of Medical and Health Sciences, University of Auckland, Auckland, New Zealand; 2 Department of Renal Medicine, Middlemore Hospital, Counties Manukau Health, Auckland, New Zealand; 3 Baxter Healthcare (Asia) Pte Ltd, Singapore, Singapore; 4 Department of Medicine & Therapeutics, Prince of Wales Hospital, The Chinese University of Hong Kong, Shatin, Hong Kong, China; 5 Research Division of Chronic Kidney Disease and Dialysis Treatment, Tohoku University Hospital, Sendai, Japan; 6 Nephrology Department, St Lukes International Hospital, Tokyo, Japan; 7 Department of Nephrology, Waikato District Hospital, Hamilton, New Zealand; 8 Nursing and Health Science, Eastern Institute of Technology, Hawke’s Bay, New Zealand; 9 Institute of Nephrology, Guangdong Medical University, Dongguan, Guangdong China; 10 Department of Nephrology, The First Affiliated Hospital, Sun Yat-sen University, Guangzhou, China; 11 Department of Medicine, University of Otago Christchurch, Christchurch, New Zealand

**Keywords:** Hemodialysis, Systematic review, Peritoneal dialysis, Mortality, Cause-specific mortality

## Abstract

**Background:**

At least 2.6 million adults and children receive dialysis treatment for end-stage kidney disease (ESKD) worldwide. The large majority of these receive hemodialysis (HD), while the remaining receive peritoneal dialysis (PD). Peritoneal dialysis may be associated with similar mortality outcomes as HD, and patient-reported outcomes are potentially increased with PD. Existing evidence for the mortality associated with PD was summarized over 20 years ago, and there has been greater marginal improvement in survival with PD relative to HD since that time. It is therefore timely to reexamine the question of differential mortality by modality and summarize evidence from more contemporary practice settings.

**Methods/design:**

Electronic databases will be systematically searched for publications that report the association between dialysis modality (HD or PD) with death from any cause and cause-specific death in incident patients with end-stage kidney disease. The database searches will be supplemented by searching through citations and references and consultation with experts. Studies published before 1995 will be excluded. Screening of both titles and abstracts will be done by two independent reviewers. All disagreements will be resolved by an independent third reviewer. A quantitative meta-analysis of effect sizes and standard errors will be applied.

**Discussion:**

Our systematic review will update previous evidence summaries and provide a quantitative and standardized assessment of the contemporary literature comparing HD and PD including published and unpublished non-English studies from greater China, Taiwan, and Japan. This review will inform shared decision-making around initial dialysis modality choice and jurisdiction-level considerations of dialysis practice.

**Systematic review registration:**

PROSPERO CRD42018111829

## Introduction

### Rationale

At least 2.6 million adults and children receive dialysis treatment for end-stage kidney disease (ESKD) worldwide, and a substantial number remain without access to dialysis care [1]. Globally, close to 90% of long-term dialysis patients receive hemodialysis (HD) with the remaining receive peritoneal dialysis (PD). The distribution of dialysis modality, however, varies widely by health jurisdiction and country. Peritoneal dialysis may be associated with similar mortality outcomes as compared to HD [2–4], although patient-reported outcomes are potentially increased with PD (e.g., patient satisfaction [5–7], life participation [8], treatment flexibility and intrusiveness [9], self-management [7], some domains of health-related quality of life [10, 11], and health utility [12–14]). Importantly, for most health care systems, PD is less expensive to provide than HD, and economic evaluations suggest improved productivity and societal outcomes with greater use of PD [15–20].

There are several potential barriers to the adoption of PD, some of which relate to clinician attitudes towards its safety and efficacy relative to HD. Where there is strong clinical belief, adoption of PD is higher irrespective of financial or infrastructure constraints [21–24]. When there is equipoise, the addressing of barriers becomes critical to increased adoption [25]. Clinician uptake of PD may of course depend on non-medical factors, such as financial incentives [26], clinical culture and disposition among peers [27], and familiarity and confidence in achieving the outcomes that are seen in centers of excellence [28, 29].

Existing evidence for the mortality associated with PD was summarized over 20 years ago, when outcomes associated with PD were accepted to be inferior to HD [30, 31]. Since those reviews, new evidence has emerged on outcomes from Australia and New Zealand [32], Canada [33, 34], the USA [35, 36], the Netherlands [37], Denmark [38], Taiwan [39], and Korea [40]. These studies indicate a greater marginal improvement in survival with PD relative to HD over the last two decades, with recent health technology assessments suggesting that the conclusions from the older studies may no longer be valid [16, 41, 42]. It is therefore timely to re-examine the question of differential mortality by modality and summarize evidence from more contemporary practice settings.

We will conduct a systematic review to evaluate the association between dialysis modality (HD or PD) with death from any cause and cause-specific death in incident patients with end-stage kidney disease. The primary outcome will be death from any cause.

## Methods

We will conduct a systematic review with meta-analysis according to reporting standards [43]. Literature searchers, identification of eligible studies, data extraction, and bias assessment will be undertaken independently by at least two researchers.

### PICO tables and eligibility criteria

The PICO criteria were agreed by the review researchers and defined as follows. Participants of eligible trials and studies will be adults and children with incidence of end-stage kidney disease starting long-term dialysis treatment. The intervention will consider any type of PD and its variants (continuous ambulatory PD (CAPD), automated PD (APD)/continuous cyclic PD (CCPD), (nocturnal) intermittent PD (IPD/NIPD), tidal PD (TPD), or continuous flow PD (CFPD)). The comparison will consider HD and its variants (hemofiltration, hemodiafiltration, acid-free biofiltration). We will exclude studies evaluating combined HD and PD strategies, or where the hemodialysis comprises intensive dialysis (i.e., greater than 3.5 times per week, or greater than 6 h per treatment [44]). The primary outcome will be death from any cause.

Eligible studies and trials will include published or unpublished reports in any language that assess associations between PD and HD with the outcome of interest. We will include randomized controlled trials and quasi-RCTs and prospectively or retrospectively recruited longitudinal cohort studies. We will exclude studies published before 1995. Narrative reviews and health technology assessments related to the topic will be retained to investigate their references for further eligible studies.

### Literature search

We will identify studies and trials from a highly sensitive literature search to identify all published and unpublished studies (Appendix in Table 1). The following databases will be searched from inception to present: MEDLINE; Embase; CENTRAL; Ichushi-Web; clinical trials registries (ClinicalTrials.gov, International Clinical Trials Registry Platform Search Portal (ICTRP), EU Clinical Trials Register, Japan Primary Registries Network (JPRN), China’s Clinical Trial Registry (ChiCTR)); China National Knowledge Infrastructure (www.cnki.net); Chongqing VIP Information Co., Ltd., formerly known as Database Research Center under Chongqing Branch of Institute of Scientific & Technical information of China (CB-ISTIC, www.wanfangdata.com.cn); HK government library (https://www.hkpl.gov.hk/en/e-resources/e-databases/keyword/e-database/all/1); Hyread full-text database of Taiwan (http://www.hyread.com.tw/hyreadnew/); Ericdata Higher Education Knowledge Base (http://www.ericdata.com/); Taiwan Journal Papers Index System (http://readopac.ncl.edu.tw/nclJournal/index.htm); TAO Taiwan Academic Online (http://tao.wordpedia.com/); and Ariti library (http://www.airitilibrary.com/).

We will search manually for additional studies by cross-checking the reference lists of all included primary studies and lists of relevant systematic reviews. In addition, study authors and experts will be contacted for additional studies. The search strategy will be developed by the research team in collaboration with an experienced librarian and checked by a referee according to the Peer Review of Electronic Search Strategies (PRESS) guidelines. The search strategy is shown in Appendix. Search results will be managed using Endnote (Clarivate Analytics, Philadelphia, PA).

### Study selection

The title and abstract of each article will be screened and assessed against predefined inclusion criteria by two independent reviewers. Full texts of all potentially relevant articles will then be assessed for inclusion by two reviewers independently. Disagreements will be resolved through discussion and consensus or consulting a third person. The corresponding authors of eligible articles will be contacted for clarification where necessary. We will record the reasons for exclusion and report the study selection process using the PRISMA flow diagram. A list of excluded studies will be provided.

### Data extraction

A standardized data extraction sheet will be designed and tested. Two reviewers will independently extract data from the included studies. Any disagreements will be resolved through discussion and consensus or by involving a third reviewer. Where necessary, studies will be translated before assessment and data extraction.

The following data will be extracted: study characteristics (design, sample size, duration of follow-up, number of participants randomized/included in the analysis); participant characteristics—demographics (age, sex), relevant medical conditions, and cause of ESKD; presence and extent of adjustment for co-variates (age, sex, diabetes mellitus); sub-modality of PD; and sub-modality of HD death from any-cause.

In case outcome data are missing, we will contact study authors and request the data. For prospective and retrospective studies, the most adjusted values for effect size will be extracted.

### Risk of bias assessment

We will use the Cochrane tool to assess study risk of bias in randomized and quasi-randomized trials. For each assessment, we will provide support for judgment. For non-randomized studies, the Newcastle-Ottawa Scale will be used. Items will be rated as low, high, or unclear risk of bias. The following domains will be assessed: representativeness of exposed cohort, ascertainment of exposure, statistical methods, outcomes of interest defined a priori (outcomes reporting bias), assessment of outcomes, and follow-up times for outcomes and attrition [45]. Any disagreements will be resolved through discussion and consensus. If necessary, we will involve a third reviewer.

### Data analysis

For prospective and retrospective cohort studies, we will summarize the adjusted risk ratios (relative risk, hazard ratio, odds ratio) for PD versus HD as reported by the studies or calculated for dichotomous outcomes using DerSimonian and Laird random effects meta-analysis. A summary risk estimate will be reported together with a 95% confidence interval. When individual studies report results separately for multiple subgroups of patients, we will extract results for each cohort to include in the meta-analysis. The results for each cohort within a study will be combined using fixed effect meta-analysis before being entered into the overall meta-analytical model. Results for observational studies and trials will be summarized separately.

Clinical and statistical heterogeneity between studies will be assessed by two reviewers. We will evaluate for heterogeneity using the *I*^2^ statistic and consider the *I*^2^ thresholds of < 25%, 25–49%, 50–75% and > 75% to represent low, moderate, high, and very high heterogeneity, respectively. Given the likelihood of clinical or statistical heterogeneity, we will apply a random-effect model. Analyses will be conducted using Stata IC 14/15 (Statacorp, College Station, TX).

Potential sources of statistical heterogeneity will be evaluated through subgroup analyses. If possible, we will undertake subgroup analyses according to age (children, adults), duration of follow-up (6 months, 1 year, 2 years), era of study (> 2000, 2000–2010, > 2010), and the type of country of study according to its economy and capital markets (advanced, developing [46]). Effect modification by age, gender, and diabetes will be ascertained by meta-regression of study-level summary data and, depending on those results, explored in subgroups according to cut points suggested by the visual inspections of fitted models. Where possible, we will conduct the following analyses to determine if results are sensitive to the influence of fixed-effect model versus random-effect model assumptions; the inclusion of studies at high risk of bias (the overall risk will be considered high if any of the domains of the Cochrane Risk of Bias tool are judged to be at high risk of bias for RCTs and if the comparability of cohorts is not enhanced by design or analyses that adjust, stratify, or match for age and diabetes); the inclusion of publications that include deaths up to 90 days (including the interim or short-term HD patients who have very high mortality due to elements unrelated to dialysis); and studies using an as-treated framework (“did the exposure that the patient actually receive affect mortality?”) (e.g., [47–50]), as opposed to an intention-to-treat framework (“did exposure that the patient initially receive affect mortality, irrespective of subsequent changes that occurred along the way?”) [51].

### Small study effects

If there are 10 or more studies included in the meta-analysis, we will investigate small study effects using funnel plots and Egger’s test.

### Level of evidence

The confidence that may be placed in the summary estimates will be evaluated using the Grading of Recommendations Assessment, Development, and Evaluation (GRADE) tool [52]. The following domains will be considered: risk of bias/study limitations, directness of evidence (generalizability), consistency of prognostic estimates among studies, and precision (width of confidence interval and impact on clinical significance). The quality of the body of evidence will be assessed by two reviewers independently. The GRADE system specifies four levels of certainty, namely, high quality (where further research is very unlikely to change our confidence in the estimates of effect), moderate (where further research is likely to have an important impact on our confidence in the estimate of effect and may change the estimate), low quality (where further research is very likely to have an important impact on our confidence in the estimate of effect and is likely to change the estimate), and very low quality (where any estimate of effect is very uncertain) evidence.

## Discussion

Our systematic review will update previous evidence summaries and provide a quantitative and standardized assessment of the contemporary literature comparing PD with HD including non-English studies from China, Taiwan, and Japan. This review will inform shared decision-making around initial dialysis modality choice and jurisdiction-level considerations of dialysis practice. Our review does not address the important outcome of quality of life. This would require a different technical scope, firstly due to the varying expressions for quality of life in the dialysis literature [12, 13] and secondly due to the different approaches to summarizing them [10, 11, 14]. As such, quality of life is beyond the scope of this review, although it is a high priority for future study with appropriate planning and resourcing.

## Presenting and reporting the results

This protocol adheres to the Preferred Reporting Items for Systematic Review and Meta-Analysis-Protocols (PRISMA-P) [43].

## Appendix

**Table 1:** Electronic search strategies

Database	Search terms
CENTRAL	MeSH descriptor Renal Replacement Therapy, this term only MeSH descriptor Hemofiltration explode all trees MeSH descriptor Hemodialysis, Home, this term only MeSH descript Renal Dialysis, this term only hemofiltrat* or haemofiltrat* or hemodial* or haemodial*:ti,ab,kw hemodiafiltrat* or hemodiafiltrat*:ti,ab,kw (HD or HDF or HF or AFB or RRT):ti,ab,kw (#1 OR #2 OR #3 OR #4 OR #5 or #6) “peritoneal dialysis”:ti.ab.kw (CAPD or CCPD or APD or PD or IPD or NIPD or TPD or CFPD):ti.ab.kw (#9 OR #10) (#8 AND #11)
MEDLINE	Renal Replacement Therapy/ Renal Dialysis/ exp. Peritoneal Dialysis/ peritoneal dialysis.tw. (PD or CAPD or CCAP or APD or IPD or NIPD or TPD or CFPD).tw. Hemodialysis, Home/ (hemodialysis or haemodialysis).tw. (HDF or HD or HF).tw. (hemodial$ or hemodial$).tw. Or/1-9 Cohort studies/ Incidence.tw. Mortality/ Follow-Up Studies/ Pronos$.tw. Predict$.tw. Course.tw. Survival Analysis/ Or/11-18 and/10,19
Embase	Peritoneal Dialysis/ Continuous Ambulatory Peritoneal Dialysis/ peritoneal dialysis.tw. (PD or CAPD or CCPD or APD or IPD or NIPD or TPD or CFPD).tw. exp. Renal replacement therapy/ hemodialysis/ home dialysis/ (hemodialysis or haemodialysis).tw. Or/1-8 Cohort Analysis/ Incidence/ Mortality/ Follow Up/ Survival/ Prognosis/ Prediction/ Or/10-16 And/9,17

## Abbreviations

APD: Automated peritoneal dialysis
CAPD: Continuous ambulatory peritoneal dialysis
CB-ISTIC: Chongqing Branch of Institute of Scientific & Technical information of China
CCPD: Company Profiles Database
CCPD: Continuous cyclic peritoneal dialysis
CFPD: Continuous flow peritoneal dialysis
ChiCTR: China’s Clinical Trial Registry
ESKD: End-stage kidney disease
GRADE: Grading of Recommendations Assessment, Development and Evaluation
HD: Hemodialysis
ICTRP: International Clinical Trials Registry Platform Search Portal
IPD: Intermittent peritoneal dialysis
JPRN: Japan Primary Registries Network
NIPD: Nocturnal intermittent peritoneal dialysis
PD: Peritoneal dialysis
PET: Peritoneal equilibration test
RCT: Randomized controlled trial
TAO: Taiwan Academic Online
TPD: Tidal peritoneal dialysis
